# Microbiota in Dung and Milk Differ Between Organic and Conventional Dairy Farms

**DOI:** 10.3389/fmicb.2020.01746

**Published:** 2020-07-28

**Authors:** Sofia I. F. Gomes, Peter M. van Bodegom, Maaike van Agtmaal, Nadejda A. Soudzilovskaia, Monique Bestman, Elza Duijm, Arjen Speksnijder, Nick van Eekeren

**Affiliations:** ^1^Institute of Environmental Sciences, Leiden University, Leiden, Netherlands; ^2^Louis Bolk Institute, Bunnik, Netherlands; ^3^Naturalis Biodiversity Center, Leiden, Netherlands

**Keywords:** dung, grassland, microbiome, milk, silage, soil

## Abstract

Organic farming is increasingly promoted as a means to reduce the environmental impact of artificial fertilizers, pesticides, herbicides, and antibiotics in conventional dairy systems. These factors potentially affect the microbial communities of the production stages (soil, silage, dung, and milk) of the entire farm cycle. However, understanding whether the microbiota representative of different production stages reflects different agricultural practices – such as conventional versus organic farming – is unknown. Furthermore, the translocation of the microbial community across production stages is scarcely studied. We sequenced the microbial communities of soil, silage, dung, and milk samples from organic and conventional dairy farms in the Netherlands. We found that community structure of soil fungi and bacteria significantly differed among soil types, but not between organic versus conventional farming systems. The microbial communities of silage also did not differ among conventional and organic systems. Nevertheless, the dung microbiota of cows and the fungal communities in the milk were significantly structured by agricultural practice. We conclude that, while the production stages of dairy farms seem to be disconnected in terms of microbial transfer, certain practices specific for each agricultural system, such as the content of diet and the use of antibiotics, are potential drivers of shifts in the cow’s microbiota, including the milk produced. This may reflect differences in farm animal health and quality of dairy products depending on farming practices.

## Introduction

Due to the intensification of production, the dairy industry is confronted with problems including water quality, energy consumption, greenhouse gas emission, loss of biodiversity, antibiotic resistance, and animal health worldwide (FAO, 2006; Janzen, 2011). To improve agro-ecological systems and enhance on-farm self-regulating processes of the agro-ecosystem, organic farming and, more recently, nature inclusive or restoration farming are being increasingly promoted (Gomiero et al., 2011; Erisman et al., 2016). Contrary to conventional farming, organic agriculture prohibits the use of artificial fertilizers, pesticides, and herbicides and limits the use of antibiotics. In addition, the cow’s diet in organic farms contains less concentrates and maize silage (Sundrum, 2001). Due to the differences in management between these two agricultural systems, cows from organic and conventional farms are subject to distinct environments, which potentially affect their microbiota composition.

Despite the lack of widely accepted consensus on how to measure the sustainability of such agricultural systems, it is assumed that organic farming is more sustainable than conventional, although more in-depth studies are urged (Fess and Benedito, 2018). In the dairy farm production cycle, each production stage can influence the microbial composition of the subsequent one (Doyle et al., 2017). Therefore, the involvement and potential translocation of microbial communities across production stages is crucial for the understanding of the source of microbes and hence its subsequent impacts. Multiple production stages should be considered. First, we have the soils that produce the fodder for dairy cattle. Previous studies have shown that soils from organic and conventional arable farming methods harbor distinct microbial communities (Hartmann et al., 2015), suggesting that organic agriculture has positive effects on the soil microbial diversity and composition (Lupatini et al., 2017). Soil microbial diversity and general soil health directly affect fodder productivity and composition (Van Der Heijden et al., 2008). The grass silage fed to the cows during the winter months is usually dominated by lactic acid bacteria, highly selective of the fermentation process (McDonald, 1981) whose diversity depends on the substrate and origin (Pang et al., 2011). The substrate and provenance will depend on the composition of the fodder from which the silage is being made, fertilization, harvesting procedures, the contamination with soil and the use of silage additives. Consequently, depending on silage origin (organic vs. conventional), the microbiota of silage is likely to have a differential impact on the microbiota of the farm animals, such as its dung and ultimately the milk as a final product.

The internal microbiota composition of mammals, such as cows, is often related to their overall health. For example, it has been shown that the gut microbiota has the potential to modulate the host’s immune system, development, and physiology (Sommer and Bäckhed, 2013). For cows, the effect of different diets on the gut bacterial composition (De Menezes et al., 2011; Thoetkiattikul et al., 2013; Faulkner et al., 2017) and the microbiota characterization along with the incidence of certain diseases, such as mastitis (Zhang et al., 2015; Derakhshani et al., 2018; Ma et al., 2018), have been the important research foci. Diet is particularly relevant in shaping the microbiota of dairy cows, since the animals are under the pressure of producing large amounts of milk, which requires a high energy and nutritional demand (Sundrum, 2001). In intensive dairy production systems, animals receive a high proportion of grains and related by-products as concentrates in the ration, which have been reported to greatly influence the composition of the gastrointestinal microbiota (Dias and Ametaj, 2017). These differences in the gastrointestinal microbiota can potentially reflect modifications in the milk microbial composition (Verdier-Metz et al., 2009). The composition of the diet has also been suggested to influence milk production and composition (Tong et al., 2018) and associated milk microbiota (Zhang et al., 2015). In addition, the microbiota in the milk may also depend on the environment of the dairy farm (Zhang et al., 2015), due to the local potential sources of contamination such as teat surface or milking instruments (Verdier-Metz et al., 2009; Doyle et al., 2017).

In previous studies, microbial communities, mainly focusing on bacteria, have been assessed for separate individual production stages of a dairy farm, without evaluating the entire farm cycle concurrently. Thus, such studies do not provide an answer to the question on how distinct management practices affect the farm microbiota via transfer of microbial communities through the production stages. In this study, we aim to compare the microbiota (fungi and bacteria) of dairy farms under organic and conventional management practices on sandy and peat soils, testing the overarching hypothesis that farm environments – organic versus conventional – affect the entire dairy farm production cycle from the soil, the locally produced silage, the gastrointestinal microbiota of cows as reflected in the dung until finally the milk. Such potential impacts – as observed in the cow’s microbiota – may be indicators of cow’s health or eventually reflect particular properties of milk quality and productivity. Understanding whether the farm practices under organic and conventional agricultural systems influence the cow’s microbiota, and how, is therefore required to determine sustainable strategies for the management of dairy farms.

## Materials and Methods

### Site Description and Sampling

Ten pairs of organic and conventional dairy farms in the Netherlands were selected, of which five pairs were situated on sandy soils, while the other five pairs were situated on peat soils. Paired organic and conventional farms were neighboring farms within a distance of 500 m on the same soil type. To further avoid confounding impacts, and allowing to treat farms as replicate in our analysis, we ensured that each farm had around 70 lactating cows and that the barns of all organic and conventional farms had a stable with cow cubicles and slatted floors. The samples collected in this study followed the management practice of the dairy farmer (organic or conventional) and were not actively imposed on the animals.

On each farm, samples were taken from the soil on a pasture close to the farm, from the grass silage which was fed to the dairy cows on the day of sampling, from fresh dung collected directly from the cows, and the bulk tank milk. The measurements in feces, which were collected from spontaneous defecations from five random cows, and the milk from bulk tank milk were also non-invasive on the animals. Sampling was done within one week from March 7 to 13, 2018, when cows were being kept exclusively indoors during winter, to exclude grazing differences between farms. The sampling protocol varied for each production stage. Pre-processing of samples taken from different production stages was conducted each day immediately after sample collection.

From the soil, five cores (ø of 2.3 cm) of 0–10 cm depth were taken in a pasture near the farm and combined in a composite sample (see Supplementary Methods S1 for overview of soil properties). The grass silage was sampled with an auger (ø of 3.0 cm and 100 cm long) from the center of the silage bin from where the cows were fed at the time of sampling (see Supplementary Methods S2, S3 for overview of ration and silage properties). For DNA extraction, silage was ground with a sterile mortar and pestle with liquid nitrogen, and 250 mg of the grinded silage was transferred to the lysis solution from the NucleoMag 96 Plant Kit. The dung sample resulted from pooling equivalent amounts of feces collected from spontaneous defecations from five random cows which was then homogenized by stirring. A subsample of 250 mg was transferred to the lysis solution from the Powersoil microbead tubes. Finally, the milk was sampled from the bulk tank to represent the complete herd of each farm (see Supplementary Methods S4 for overview of milk properties). The milk samples for DNA extraction were collected in two 50 ml sterile falcon tubes and centrifuged at 5,400 × *g* for 30 min at 4°C. The fat layer was carefully removed, and the supernatant was decanted. The resulting pellets were washed twice using sterile PBS and centrifuged at 14,000 × *g* for 1 min and then pooled. Approximately 250 mg of pellet was dissolved in the lysis solution from the Powersoil microbead tubes. The pre-processed samples in the lysis solution from the extraction kit were kept at −20°C until sampling was complete and processed immediately after. All equipment for sampling soil, silage, dung, and milk collection was surface sterilized with 90% ethanol in between collections.

### DNA Extraction and Sequencing

DNA was extracted from soil, dung, and milk using the DNeasy Powersoil Kit (Qiagen, Venlo, Netherlands) and from silage using the NucleoMag 96 Plant Kit (Macherey-Nagel Gmbh & Co., Düren, Germany), following the manufacturer’s protocols. The ITS2 region was targeted to obtain the fungal communities using the fITS7/ITS4 primers (White et al., 1990; Ihrmark et al., 2012) containing an Illumina adapter overhang, and a fragment from the V4 region of the 16S rRNA gene was amplified to obtain the bacterial communities using the standard methods developed by the Earth Microbiome Project with the primers 515F/806R (Caporaso et al., 2010) containing an Illumina adapter overhang. PCR products were purified using 0.9 × NucleoMag NGS Clean-Up and Size Selectbeads (Macherey-Nagel, Düren, Germany) according to the manufacturer’s instructions. The Illumina Nextera XT adaptors were added to the amplicons using a second 20 ul PCR containing 8 cycles (Illumina, San Diego, CA, United States). The concentration of these individual indexed amplicons was measured with the QIAxcel using the DNA Screening Kit (Qiagen, Venlo) and was normalized and pooled equimolar for fungi and bacteria in individual libraries using the QIAgility robot (Qiagen, Venlo). The two pools had a final clean up with 0.9 × NucleoMag NGS Clean-Up and Size Selectbeads (Macherey-Nagel, Düren, Germany). The quality and quantity of these two pools were checked on the Bioanalyzer using a high sensitivity chip (Agilent). Sequencing was done in two runs by Illumina MiSeq platform using the paired-end 300 bp kit (BaseClear, Netherlands).

### Microbial Community Analysis

Raw sequences of both fungi and bacteria were paired based on a minimum overlap of 30 nucleotides, and the primers were trimmed. Subsequently, sequences were filtered discarding sequences with expected error >1. The quality-filtered sequences were denoised using the UNOISE3 algorithm (Nearing et al., 2018) to create zero radius operational taxonomic units (Zotus). The Zotus are exact sequence variants which provide a finer resolution in the taxa discovered than the traditional clustering methods where clusters of sequences are determined based on a fixed dissimilarity threshold (Callahan et al., 2017). Putative chimeras were removed. All the above steps were performed using USEARCH v.11 (Edgar, 2010). To remove spurious counts due to cross-talk (assignment of reads to a wrong sample), we removed all Zotus represented by <0.02% of reads in each sample, being even more conservative than previous error estimates (Bokulich et al., 2013). Both data on relative abundance and presence–absence of microbial Zotus were used in the analysis. Raw sequences have been deposited in the Short Read Archive of NCBI under the project number PRJNA627834.

The fungal Zotus were assigned to taxonomic groups using the Blast algorithm based on pairwise similarity searches queried against the curated UNITE+INSD fungal ITS sequence database (version 7.2, released on October 10, 2017), which contains approximate species-level OTUs assignments to representative sequences of fungi (Kõljalg et al., 2013). The Zotus were retained if they had >80% similarity and >150 bp pairwise alignment length to a fungal Species Hypothesis from the fungal database. The bacterial Zotus were assigned to taxonomic groups by querying against the SILVA database (Quast et al., 2013) containing curated 16S sequences of bacteria.

### Microbial Community Diversity Analysis

The number of reads was highly variable within the individual production stages’ datasets. Thus, to explore the general patterns of microbial diversity among production stages (soil, silage, dung, and milk), the Zotu tables were resampled to a depth of 2,500 for fungi and 1,000 for bacteria (see rarefaction curves in Supplementary Figure S1). For those samples that yielded a lower number of reads than the depth selected, we ran the subsequent analysis twice, by including and excluding these samples. The significance of the results remained unchanged; thus, we present the results for the entire dataset for completeness. Samples with <200 reads were removed, resulting in removing one soil, one silage, and one milk sample in the fungi dataset and one soil, one milk, and two dung samples in the bacteria dataset.

The phylogenetic species variability (PSV) was calculated as a measure of alpha diversity, using the *picante* R package (Kembel et al., 2010). This measure is statistically independent of species richness, thus less biased than the Faith’s phylogenetic diversity. The PSV tends to approach one when the communities within one sample are unrelated, and it approaches zero as taxa in the community become more related (Kembel et al., 2010). Phylogenetic relationships among Zotus were calculated based on alignment of fungal ITS and bacterial 16S generated with MAFFT v. 7.388 (Katoh and Standley, 2013) and tree construction using RaxML v. 8.2.12 with the GTRCAT model (Stamatakis, 2014). Phylogenies were transformed into ultrametric trees using PATHd8 (Britton et al., 2007). Pairwise distance between taxa was computed using *cophenetic.phylo* in the *ape* R package (Paradis and Schliep, 2019). The differences in PSV according to production stages and agricultural system within production stages were assessed using Kruskal–Wallis in combination with Dunn’s test as a *post hoc* test with Benjamini-Hochberg corrections for multiple comparisons, using the *dunn.test* R package (Dinno, 2017).

### Impact of Agricultural System

To display community composition differences between agricultural systems and soil type, non-metric multidimensional scaling (NMDS) was performed with Bray–Curtis dissimilarities, using the *vegan* R package (Oksanen et al., 2015). Taxa plots were generated to visualize the relative abundances of main families of fungi and bacteria within production stages.

The effect of agricultural system (organic vs. conventional) and soil types (sand vs. peat) on the microbial community composition was estimated through a model-based approach to analyze multivariate data (Warton et al., 2015) using the function *ManyGlm* incorporated in the *mvabund* R package (Wang et al., 2012). Our data presented a strong mean–variance relationship (not shown), for which model-based approaches perform better than distance-based approaches (Wang et al., 2012). We used the *ManyGlm* with a negative binomial distribution for the abundance data and a binomial distribution for the presence–absence data. With the *ManyGlm*, a model is fit to each taxon and the log-likelihood ratio (LR) of each model is summed to create an overall sum-of-LR that can be used as a test statistic via randomization. The model structure was similar throughout the analysis of the four production stages (soil, silage, dung, and milk), including the agricultural system nested within pair, and soil type as fixed categorical factors, with the interaction between agricultural system and soil type included. The composition of fungi and bacteria was analyzed separately. Examination of the residual plots from the *ManyGlm* showed no clear patterns indicating that the models were appropriate. Significance of the models was calculated using 999 resampling iterations via PIT-trap resampling method (Warton et al., 2017). To determine which taxa contributed the most to the shifts in community composition, the individual contribution of each Zotu to the overall sum-of-LR was calculated, and *P-*values were adjusted with Benjamini–Hochberg corrections for multiple testing. In addition, we applied a linear discriminant analysis (LDA) coupled with effect size measurements (LEfSe), which performs a nonparametric Wilcoxon sum-rank test followed by LDA analysis (Segata et al., 2011) to assess differentially abundant taxonomic groups (at higher taxonomic ranks than the individual Zotus) most likely to explain differences between agriculture systems (included in the analysis as classes), and subsequently using a set of pairwise tests among the two soil types (included in the analysis as subclasses). To further investigate a potential link between diet composition and cow’s microbiota (dung and milk), and also between milk properties, including milk productivity, and its microbial communities, we run multiple *ManyGlm* models for each fungal and bacterial community matrices (see Supplementary Methods S5).

## Results

### Taxonomic Assignment

#### Fungi

A total of 1,727 fungal Zotus was represented by 2,974,303 quality-filtered reads, with a mean of 38,688 and standard deviation of 17,340 reads per sample. Ascomycota (86.28% of all soil, 84.52% of all silage, 91.68% of all dung, and 72.13% of all milk reads) and Basidiomycota (10.59% of all soil, 15.01% of all silage, 7.28% of all dung, and 18.89% of all milk reads) were the most dominant phyla across all production stages. Only one Zotu belonging to the family Aspergillaceae was shared among all dung samples across farms, and none was shared among all the milk samples. Predominant fungal families present in the dung samples were Aspergillaceae (24%), Ascosphaeraceae (8.13%), and Saccharomycetaceae (6.20%); and those present in the milk samples were Aspergillaceae (5.74%), Cyphellophoraceae (4.86%), Hypocreaceae (2.90%), Microascaceae (2.62%), and Pyronemataceae (2.21%).

**FIGURE 1:**
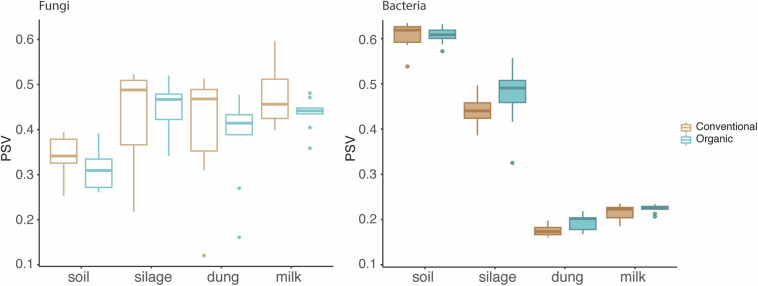
Phylogenetic diversity of fungi and bacteria in the four production stages – soil, silage, dung, and milk – measured with the phylogenetic species variability (PSV). The line that divides the boxplots represents the median value.

The phylogenetic species variability PSV differed between the four production stages (chi-square = 26.98, *df* = 3, *P* < 0.01). Soil (*n* = 19) had significantly less diverse fungal communities compared to silage (*n* = 19), dung (*n* = 20), and milk (*n* = 19). Production stages were not significantly different between each other in terms of phylogenetic diversity (Figure 1 and Supplementary Table S4). The phylogenetic diversity of fungal communities was not distinguishable between agricultural systems or soil type, for any production stage (Table 1).

**TABLE 1:** Statistical results of a Kruskal–Wallis test comparing phylogenetic species variability (PSV) of sample types among the two soil types, sand and peat, and the two agriculture systems, conventional and organic.

		**Soil type**			**Agriculture system**		
		**Chi-squared**	***df***	***p*-value**	**Chi-squared**	***df***	***p*-value**
**Fungi**	Soil	0	1	1.000	2.160	1	0.142
	Silage	1.929	1	0.165	0.347	1	0.556
	Dung	1.463	1	0.227	0.966	1	0.326
	Milk	0.06	1	0.807	1.127	1	0.289
**Bacteria**	Soil	8.167	1	**<0.001**	0.167	1	0.683
	Silage	0	1	1.000	2.94	1	0.086
	Dung	1.218	1	0.270	5.897	1	**0.015**
	Milk	3.527	1	0.060	0.960	1	0.327

*P-values of ≤0.05 are considered significant and represented in bold.*

#### Bacteria

A total of 1,446 bacterial Zotus was represented by 241,425 quality-filtered reads, with a mean of 3,177 and standard deviation of 2,743 reads per sample. Firmicutes (6.25% of all soil, 57.23% of all silage, 71.65% of all dung, and 37.90% of all milk reads), Actinobacteria (28.88% of all soil, 8.27% of all silage, 2.59% of all dung, and 38.85% of all milk reads), Proteobacteria (14.44% of all soil, 34.25% of all silage, 1.14% of all dung, and 21.34% of all milk reads), and Chloroflexi (18.25% of all soil, 0.11% of all silage, and 0.14% of all milk reads) were the four dominant phyla across all production stages. Individual Zotus shared among all dung samples across farms were Bacteroidales, Christensenellaceae, Lachnospiraceae, Rikenellaceae, Ruminococcaceae, *Akkermansia*, *Paeniclostridium*, and *Roseburia*, representing 79.07% of all dung reads. Only one Zotu belonging to the family Corynebacteriaceae was present in all milk samples across farms.

The PSV was significantly different among production stages (chi-square = 67.89, *df* = 3, *P* < 0.01). Soil (*n* = 19) had the most significantly diverse bacterial communities, followed by silage (*n* = 20), milk (*n* = 19), and dung (*n* = 18) (Figure 1 and Supplementary Table S4). The phylogenetic diversity of bacterial communities in the dung was significantly different between organic and conventional farms, while the phylogenetic diversity of bacterial communities in the soil differed significantly between sandy and peat soils (Table 1). Despite that only the diversity of bacteria in the dung, as measured by PSV, was significantly higher in organic farms compared to conventional farms (Table 1 and Figure 1), we observed that in general the PSV for bacteria in organic farms is generally higher than in conventional farms.

Fungi, however, showed an opposite trend, with fungal diversity being higher in conventional than in organic farms (Table 1 and Figure 1).

### Comparison of Microbial Diversity in the Four Production Stages

**FIGURE 2:**
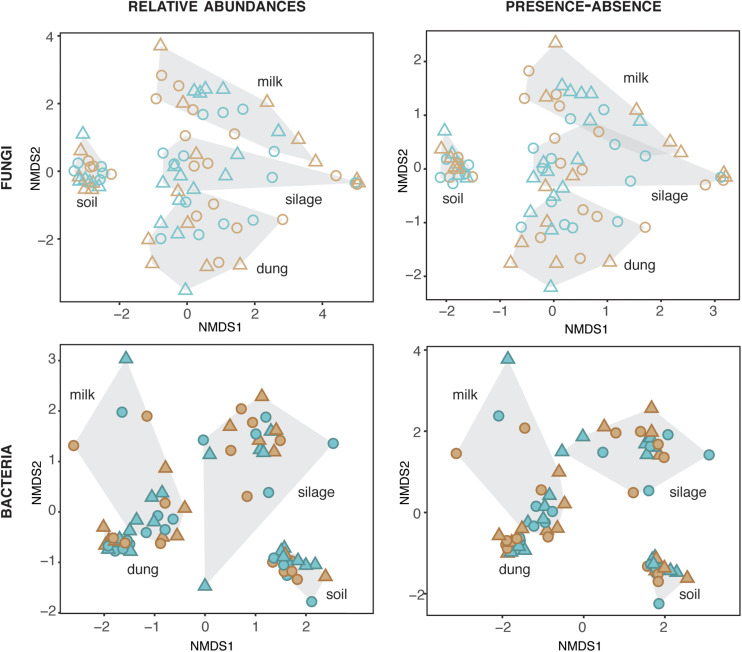
Fungal and bacterial diversity in the four production stages, assessed based on relative abundances and presence–absence datasets. Blue and brown symbols indicate organic and conventional farms, respectively.

**FIGURE 3:**
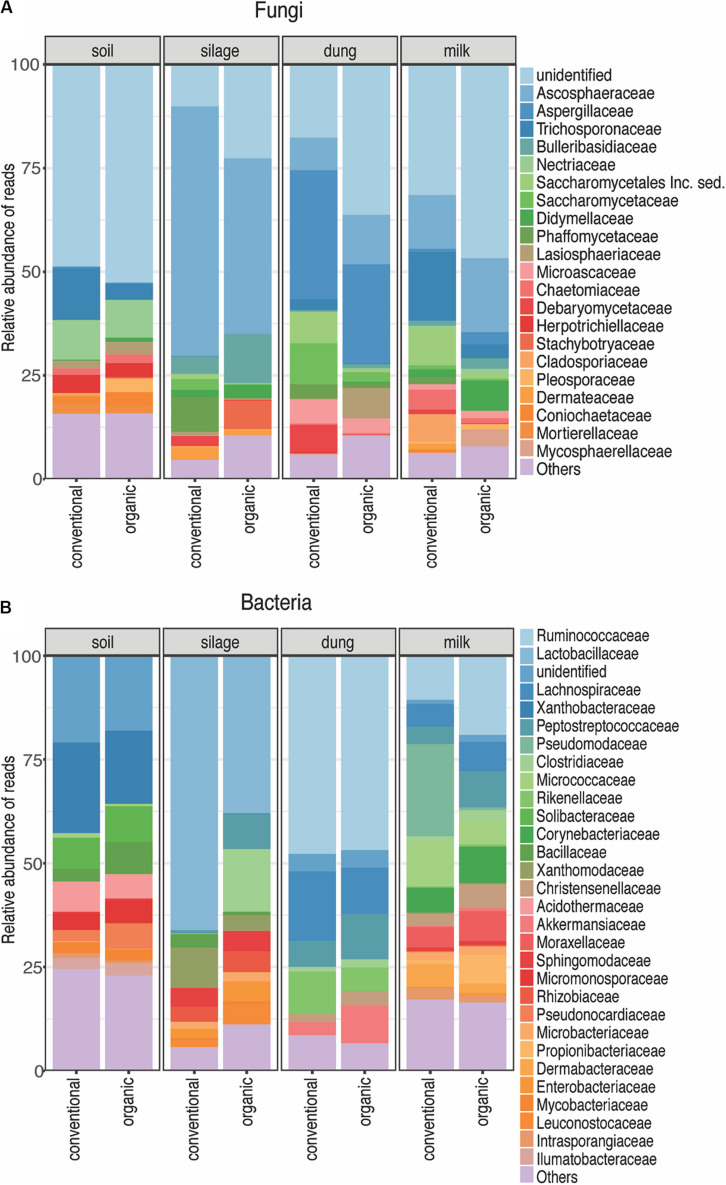
Relative abundance of each fungal **(A)** and bacterial **(B)** family present in each production stage according to agriculture systems. Sampling depth was set to 1500 in fungi and 1000 in bacteria. The Zotus that could not be identified below order level are summarized as “unclassified”. All the families represented by less than 5% of total reads were summarized in “Others”.

The composition of fungi and bacteria was structured by production stage for both relative abundances (fungi: ManyGlm deviance = 12,341, *df* = 73, P = 0.001; bacteria: deviance = 30,712, *df* = 72, *P* = 0.001) and presence–absence data (fungi: ManyGlm deviance = 12,332, *df* = 73, *P* = 0.001; bacteria: deviance = 28,847, *df* = 72, *P* = 0.001). Hence, each production stage (soil, silage, dung, and milk) was characterized by distinct microbial community compositions (Figure 2), and relative abundance of main specific families of fungi and bacteria (Figure 3 and Supplementary Tables S5, S6). The fungal communities of silage, dung, and milk were more similar among each other than those of soil, while the bacterial communities of dung and milk were more similar in comparison to those of silage and soil (Figure 2).

### Impact of Agricultural Systems on Microbial Communities and Their Potential Drivers Within Production Stages

#### Soil

**FIGURE 4:**
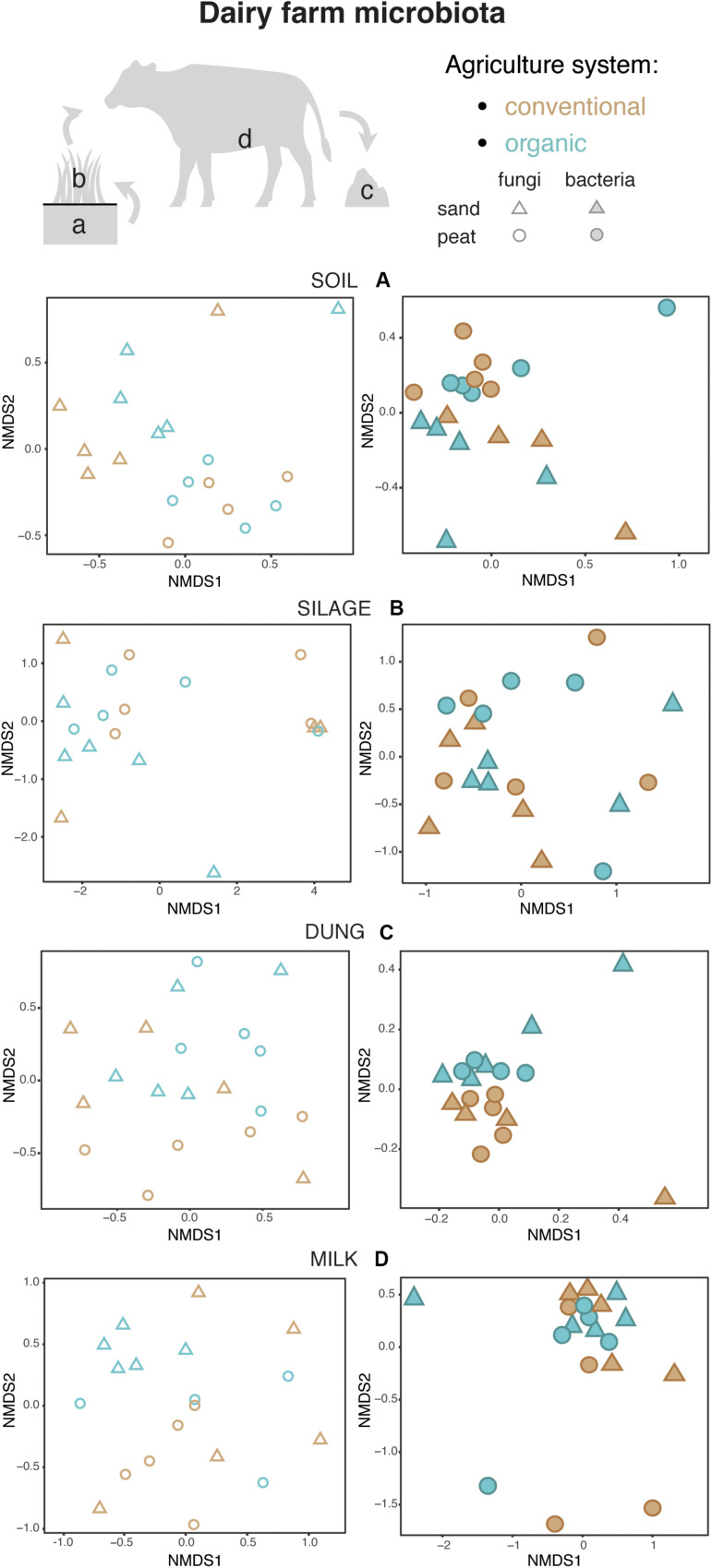
Fungal and bacterial communities’ structure in the soil **(A)**, silage **(B)**, dung **(C)**, and milk **(D)**, colored according to agriculture systems. Fungi are represented as empty symbols and bacteria with full symbols. Parallel figures based on the relative abundance dataset at the Zotu level are portrayed in Supplementary Figure S2.

Both in terms of presence–absence as well as of relative abundances, fungal and bacterial communities were not structured by agricultural system, while with presence–absence only, both fungi and bacteria were significantly structured according to soil type (Tables 2, 3 and Figure 4A). However, the LEfSe analysis showed that bacteria in the soil, such as *Dactylosporangium*, *Micromonospora*, and *Chloroflexia* were statistically associated with organic farms, while the order Tepidisphaerales was associated with conventional farms (Supplementary Figure S2). The analysis of relative abundances of soil fungi yielded a significant agricultural system by soil type interaction (Supplementary Tables S7, S8 and Supplementary Figure S3A).

**TABLE 2:** Analysis of deviance table for fungal community composition based on the presence–absence dataset within the four production stages (soil, silage, dung, and milk) according to agriculture systems (conventional vs. organic) and soil type (sand vs. peat).

		**Residual df**	***df***	**Deviance**	***P*-value**
**Soil**	Agriculture	17	1	318.4	0.462
	Soil type	16	1	920	**0.001**
	Agric: Soil	15	1	417.2	0.128
**Silage**	Agriculture	18	1	494	0.251
	Soil type	17	1	562.6	0.145
	Agric: Soil	16	1	473.7	**0.015**
**Dung**	Agriculture	16	1	1069.6	**0.020**
	Soil type	15	1	926.6	0.057
	Agric: Soil	14	1	620.6	0.151
**Milk**	Agriculture	17	1	1177	0.244
	Soil type	16	1	1286	0.205
	Agric: Soil	15	1	879	0.129

*Multivariate test statistics: log-likelihood ratio with 999 resampling iterations via PIT-trap method. Significant results (*P-*value < 0.05) are represented in bold.*

**TABLE 3:** Analysis of deviance table for bacterial community composition based on the presence–absence dataset within the four production stages (soil, silage, dung, milk) according to agriculture systems (conventional vs. organic) and soil type (sand vs. peat).

		**Residual df**	***df***	**Deviance**	***P*-value**
**Soil**	Agriculture	17	1	321	0.543
	Soil type	16	1	808.1	**0.004**
	Agric: Soil	15	1	350.5	0.053
**Silage**	Agriculture	18	1	530.8	0.156
	Soil type	17	1	367.3	0.652
	Agric: Soil	16	1	274.3	0.107
**Dung**	Agriculture	16	1	933.6	**0.046**
	Soil type	15	1	945	0.054
	Agric: Soil	14	1	501.8	**0.033**
**Milk**	Agriculture	17	1	1141.7	0.347
	Soil type	16	1	1206.3	0.275
	Agric: Soil	15	1	781.2	0.059

*Multivariate test statistics: log-likelihood ratio with 999 resampling iterations via PIT-trap method. Significant results (*P*-value < 0.05) are represented in bold.*

#### Silage

Presence–absence data suggest that fungal and bacterial communities were not structured by agricultural system or soil type (Tables 2, 3 and Figure 4B), while the relative abundance data reveal that fungal communities were significantly affected by soil type, and bacterial communities by an interaction between agricultural system and soil type (Supplementary Tables S7, S8 and Supplementary Figure S3B). The LEfSe analysis showed that the fungal order Hypocreales was statistically associated with silage samples from organic farms (Supplementary Figure S2).

#### Dung

Both fungal and bacterial communities were structured by agricultural system, with an interaction between agricultural system and soil type for bacteria for the presence–absence analysis (Tables 2, 3 and Figure 4C). According to the LEfSe analysis, only bacteria, such as *Succinivibrio*, *Prevotella*, *Aeromodales*, were statistically associated with dung samples in conventional farms (Supplementary Figure S2). The analysis of the relative abundances suggested that fungal communities were significantly structured according to agricultural system and soil type, including an interaction between the two variables, while bacterial communities differed significantly only between agricultural systems (Supplementary Tables S7, S8 and Supplementary Figure S3D). Fungal communities were significantly associated with the percentages of grass silage in the diet and the antibiotic use (Supplementary Table S9).

#### Milk

The presence–absence analysis revealed that fungal and bacterial composition were not affected by agriculture or soil type (Tables 2, 3 and Figure 4D). Yet, in terms of presence–absence, fungal communities were significantly associated with diet composition (e.g., amount of concentrates and percentage of grass silage) (Supplementary Table S9), with the use of antibiotics, and also with milk production, fat and urea milk content, while bacterial communities were associated with urea and fat milk content (Supplementary Table S10). In terms of relative abundances, fungal communities were significantly structured by agricultural system, and also soil type, including an interaction between the two variables. The LEfSe analysis showed a significant association of the fungi Dothideomycetes, Tremellomycetes, and Pleosporales with milk samples from organic farms (Supplementary Figure S2). Moreover, fungal communities were significantly associated with the use of antibiotics, amount of concentrates in the diet (Supplementary Table S9), and also with the milk production (Supplementary Table S10). Bacterial communities did not vary significantly among agricultural system or soil type (Supplementary Tables S7, S8 and Supplementary Figure S3C) but milk samples in conventional farms were statistically associated with the family Rhodobacteraceae (Supplementary Figure S2).

We did not find a statistical association between any fungal or bacterial Zotu of any production stage with agricultural systems.

## Discussion

We tested the hypothesis that dairy farm environments and soil types under organic and conventional management practices affect the microbiota throughout the different stages of the production cycle of the farm, including the soil, the locally produced silage, and the gastrointestinal microbiota of cows, reflected in the dung, and the milk. Our results show that management practices associated with the two agricultural systems did not have a significant effect on soil and silage microbiota. However, the dung and milk microbiota of cows differed between organic and conventional systems, suggesting a disconnection between the analyzed stages of the farm production cycle in terms of microbial transfer, but still an association with agricultural system. The differences reported in the cow microbiota according to agriculture practices are in line with recent studies indicating that shared environments can have a greater impact on shaping individuals microbiota than genetics (Rothschild et al., 2018). Furthermore, the intrinsic differences in diet composition and use of antibiotics between organic and conventional systems might play an important role in affecting the cow’s microbiota, as suggested in previous studies (Hagey et al., 2019).

The diversity of fungi and bacteria in all production stages – soil, silage, dung, and milk – was affected by agricultural system and soil type, but in different ways. Each production stage displayed particular taxonomic groups of fungi and bacteria, yet not a single Zotu was significantly associated with either agricultural system. In general, the most prevalent taxa among production stages were present in samples of both organic and conventional farming systems. These are probably representatives of “core” functional groups belonging to the “core” microbiome of each production stage (Turnbaugh et al., 2009). Thus, the effects observed due to the influence of agriculture practice and soil type are likely to have occurred at the taxa found at a lower abundance.

Fungal and bacterial communities in the soil were structured by soil type – but not by agricultural system – which is in agreement with the findings of Fierer and Jackson (2006) and Fierer (2017) who have shown that the edaphic properties inherent to particular soil types are the principle drivers of soil microbial composition. Previous studies found differences in organic and conventional practices, namely that the addition of organic fertilizers only instead of the addition of artificial fertilizers led to significant differences of microbial composition mainly in arable soils, of fungi (Lalande et al., 2005; Ghimire et al., 2014) and also of bacteria (Mas-Carrió et al., 2018) but that the effect of grassland is dominating the effect of system (Rutgers et al., 2008; Deru et al., 2018). The fermentation process that silage undergoes that selects for lactic acid bacteria (McDonald, 1981) most likely determined its microbial composition, as observed by the high relative abundance of Lactobacillaceae. This effect seems to be stronger than differences in management or substrate provenance between organic and conventional farms.

Previous studies have shown that diet has a direct impact on the microbiota of cattle (Zhang et al., 2015; Loor et al., 2016; Hagey et al., 2019). Accordingly, we expected to detect a direct effect of silage microbial communities from each agriculture system on the cow’s microbiota. We found no differences in the microbial community composition of silage produced by organic or conventional farms and fed to the cows in winter months. Yet, surprisingly, we detected that cow’s dung microbiota, including both fungi and bacteria composition, significantly differed between animals under organic and conventional management. The diversity of bacteria was significantly higher in cows from organic farms, which is in agreement with previous studies that found that concentrate rich diets and the use of antibiotics, characteristic practices in the conventional farms, have negative effects on the diversity of bacteria (Thomas et al., 2017; Zhang et al., 2017). Contrasting with the dung, in the milk only changes in the relative abundance of fungal communities seemed to be related to agricultural system, but not its community composition, and neither of bacteria. Thus, in our study, the cows’ microbiota was not linked to the microbial composition of the silage under organic versus conventional management practices. Instead, the fungal community composition in both dung and milk were linked to the percentage of grass silage in the diet and the use of antibiotics. Also, the amount of concentrates in the diet influenced the fungi in the dung. The higher relative proportion of maize silage and the amount of concentrates in the cow diet in conventional farming, together with the higher use of antibiotic treatments often constitute a major distinction between conventional and organic farming systems (Dias and Ametaj, 2017). In this study, we reported an increase of *Prevotella* in the dung of cows from conventional farms. These bacteria are very common in the rumen and are characteristic in the rumen of animals that have a diet rich in grains (Tajima et al., 2001). Moreover, in conventional farms we reported an increase in bacteria such as *Succinivibrio.* This could indicate that cows in conventional farms have a lower residual feed intake (Hernandez-Sanabria et al., 2012) or have a more efficient digestion than those in organic farms (VandeHaar et al., 2016). In terms of fungi, unexpectedly we found only 0.05% of the fungal reads in dung to belong to Neocallimastigaceae, a family that contains anaerobic fungi often found in the digestive track of herbivores. No taxa was significantly associated with any of the management practices, suggesting that the differences in diet have a lower influence on fungi compared to bacteria, as also reported previously (Zhang et al., 2017). Furthermore, antibiotic treatments have been shown to suppress specific bacteria in the gut and subsequently in the dung of ruminants (Holman et al., 2019). Our study thus stresses that the differences observed in the microbiota of the dung and milk of cows in organic and conventional farms do not result from translocation of the microbiota present in the diet but are linked to the content of the diet and the use of antibiotics of each farm management practice. Future studies should investigate the presence of antimicrobial resistance genes in the cow microbiota in relation to antibiotic use in dairy farms (Thomas et al., 2017).

The association of Dothideomycetes, including Pleosporales, and Tremellomycetes with milk from organic farms is surprising and unknown, since fungi belonging to these classes often include plant pathogens that grow on wood debris or decaying leaves, yet their presence have been reported in the environment of dairy farms (Mbareche et al., 2018) and in shelves used for ripening of cheese (Guzzon et al., 2017). Airborne dust microbiota and housing conditions, such as bedding, have been described to affect milk microbiota composition, because the teats of cows contact directly with the bedding material when the cows are resting (Nguyen et al., 2019; Wu et al., 2019) and the teat microbiota is a known source of contamination of the milk microbiota (Verdier-Metz et al., 2009; Doyle et al., 2017). Perhaps the reported fungi in this study occur more commonly within the environment of organic farms in particular substrates, but further research is needed to identify the source of these fungi and to evaluate their importance in the context of dairy farms. The overall composition of fungi and bacteria showed a significant association with diet composition, namely the amount of concentrates and percentage of grass silage, and also with the use of antibiotics. Interestingly, the microbial composition also varied with the urea and fat milk content, and milk production. Milk properties have been shown to indirectly shape the milk microbial community (Williams et al., 2017; Moossavi et al., 2019). Thus, our results indicate that milk properties differ between organic and conventional farms both in terms of nutritional properties as well as in terms of microbial composition.

## Conclusion

In conclusion, our study explored the potential for microbial transfer through the different production stages in dairy farms that encompass organic and conventional agricultural practices. Our results demonstrate that cow’s microbiota reflected in the dung and milk is affected by the agricultural management system, despite the fact that no differences were found in the microbial communities of soil and silage. These impacts of agricultural management systems seem primarily related to the amounts of concentrates and percentage of grass silage in the diet, and by the use of antibiotics. Given that the milk fat and urea content and milk productivity are associated with microbial (fungi and bacteria) communities in the milk, this additionally indicates that agricultural management may affect milk quality. Further research is warranted to explore the impacts of the different microbial communities associated with organic and conventional agricultural systems in the dung and milk on cow’s health, its consequences for human consumption, and the impact on sustainable production of dairy products.

## Data Availability Statement

Raw sequences have been deposited in the Short Read Archive of NCBI under the project number PRJNA627834.

## Author Contributions

SG, NS, and NE contributed to the conception and design of the study. SG, MA, MB, and NE collected the samples. ED and AS supervised the molecular work. SG, PB, and NS conducted the statistical analysis. SG wrote the first draft of the manuscript. All authors contributed to manuscript revision, read, and approved the submitted version.

## Conflict of Interest

The authors declare that the research was conducted in the absence of any commercial or financial relationships that could be construed as a potential conflict of interest.
