# Supplementary material for: Microbiota in Dung and Milk Differ Between Organic and Conventional Dairy Farms
Source: Front Microbiol. 2020 Jul 28;11:1746. doi: 10.3389/fmicb.2020.01746 (PMC7399162; doi:10.3389/fmicb.2020.01746)
Supplement: Supplementary file 1 [file Data_Sheet_1.docx]

Supplementary Material

# Methods

**Methods S1: Soil nutrient composition and extraction**

For each site, soil from the 0-10 cm bulk sample was oven-dried at 40°C prior to analysis of soil acidity (pHKCl), SOM, total nitrogen (Ntotal), total phosphate (P-Al) and potassium (K) in solution. Soil acidity of the oven-dried samples was measured in 1 M KCl (pH-KCl). SOM was determined by loss-on-ignition. For determination of Ntotal evolved gasses after incineration were reduced to N2 and detected with a thermal-conductivity detector (LECO Corporation, St. Joseph, Mich., USA). P-Al was determined using standard methods (Bronswijk et al., 2003). P avail was determined by flame photometry after soil extraction with HCl (0.1 M) and oxalic acid (0.5 M) in a 1:10 mass to volume M:V ratio, and filtration (Bronswijk et al., 2003). Soil particle analysis was done by a Beckman Coulter LS-230 laser with software version 3.01 and firmware version 2.02. Particle analysis was performed after removal of CaCO3 with 1 M HCl, and addition of de-ionized water, and of 30% H2O2 to remove organic matter, at 80-95 ͦ C.

**Soil** samples were significantly different in soil organic matter and nutrient composition according to soil type – sand vs peat – but there were no differences in soil properties between agricultural systems (see SI Table S1 for Kruskal-Wallis tests).

**Methods S2: Ration composition**

In an interview with the different farmers the antibiotics animal day doses of the year 2017, the milk production and the ration composition of February / March 2018 were registered.

Conventional farms used significantly more concentrates and maize silage compared to organic farms, while the **ration** from organic farms had significantly more grass silage. The antibiotics animal day doses were almost zero on organic farms and 1.59 on average on conventional farms (see SI Table S2 for Kruskal-Wallis tests).

**Methods S3: Silage composition**

Silage samples have been taken and analyzed by Eurofins Agro NL, Wageningen, The Netherlands with the Near Infra-Red Spectrometry.

In the **grass silage**, available energy for milk production (VEM), degraded protein balance (OEB), raw ash and organic matter digestibility (OMD%), were significantly different between agricultural systems, and crude fiber according to soil type (see SI Table S2 for Kruskal-Wallis tests).

**Methods S4: Milk properties**

Bulk tank milk was analyzed on protein, fat, lactose, urea, bacterial count en cell count by Qlip B.V. The NetherlandsBulk tank **milk** samples had no significant differences in content of protein, lactose, fat, urea, bacterial count, cell count, geo bacterial count and geo cell count, between agricultural systems or between soil types. Milk production per cow was significantly higher on conventional farms compared to organic farms (see SI Table S3 for Kruskal-Wallis tests).

**Methods S5: *ManyGlm* models to investigate the potential link between diet composition and cow’s microbiota, and also between milk properties and its microbial communities**.

We run multiple *ManyGlm* models for each fungal and bacterial community matrix. Because we were interested in determining potential drivers of the microbial community shifts between organic and conventional farms, we first tested whether the variables to include in the model (see SI, Table S2) were significantly associated with agriculture system and/ or soil type using a two-way ANOVA. The selected variables were standardized to mean = 0 and SD = 1 to avoid scaling variance issues and were assessed for collinearity with VIF < 3 and Pearson correlation < |0.7|. The order of the explanatory variables in the model was determined with *best.r.sq* which performs a forward selection, choosing their order based on how they best explain the variation in a multivariate response as measured by their R^2^.

To test for association between milk properties and microbial communities, we eliminated collinear variables from the model by calculating the variance inflation factors (VIFs) in a stepwise manner, discarding the variable with the highest VIF at each step, until all variables chosen had VIF < 3 and Pearson correlation < |0.7|. Lactose and protein contents were highly correlated (Pearson’s correlation: *r* = -0.7, *t* = -3.7, *df* = 17, *P* = 0.002), thus only one of them was kept in the models.

# Tables

**Table S1**: Properties measured from the soil, ration, silage and milk.

| **Production stage** | **Measured property** | **Agriculture system** | |
| --- | --- | --- | --- |
|  |  | **Conventional**  (mean ± sd) | **Organic**  (mean ± sd) |
| Soil | N _total_ (mg/kg) | 10153.00 ± 9130.17 | 10966.00 ± 9302.11 |
|  | P _avail_ (g/kg) | 3.82 ± 2.89 | 3.85 ± 3.11 |
|  | P _total_ (mg P_2_O_5_/100 g) | 55.00 ± 24.23 | 52.90 ± 22.07 |
|  | Organic Matter (%) | 23.08 ± 18.72 | 24.01 ± 20.06 |
|  | Clay (%) | 11.40 ± 12.04 | 10.30 ± 10.19 |
|  | Silt (%) | 12.90 ± 7.09 | 12.30 ± 3.20 |
|  | Antibiotics (animal day doses) | 1.59 ± 1.09 | 0.16 ± 0.18 |
| Ration | Concentrates (kg DM/cow/day) | 6.27 ± 1.53 | 4.02 ± 1.53 |
|  | Dry matter intake (kg DM/cow/day) | 21.02 ± 1.90 | 17.66 ± 1.98 |
|  | Maize silage (kg DM/cow/day) | 5.971 ± 2.36 | 2.44 ± 2.07 |
|  | Roughage (kg DM/cow/day) | 14.989 ± 1.30 | 13.66 ± 1.21 |
|  | % grass silage | 0.50 ± 0.13 | 0.73 ± 0.13 |
|  | % maize silage | 0.22 ± 0.15 | 0.05 ± 0.09 |
|  | % concentrates | 0.30 ± 0.06 | 0.22 ± 0.07 |
|  | Dry matter (%) | 42.52 ± 6.40 | 46.38 ± 12.00 |
| Silage | pH | 4.69 ± 0.36 | 4.68 ± 0.65 |
|  | Butyric acid | 1.21 ± 0.62 | 0.70 ± 0.24 |
|  | Acetic acid | 13.89 ± 6.53 | 13.60 ± 6.11 |
|  | Lactic acid | 44.89 ± 19.45 | 44.80 ± 25.92 |
|  | VEM | 932.33 ± 46.00 | 822.67 ± 76.17 |
|  | DVE | 58.56 ± 9.262 | 56.33 ± 10.46 |
|  | OEB | 55.11 ± 18.29 | 35.50 ± 19.013 |
|  | Raw Ash (g/kg DM) | 93.00 ± 9.14 | 138.83 ± 58.84 |
|  | OMD (%) | 77.60 ± 3.045 | 73.65 ± 2.86 |
|  | NH_3_ (% of CP) | 8.01 ± 3.17 | 6.50 ± 2.35 |
|  | Nitrate (g/kg DM) | 2.77 ± 2.34 | 1.42 ± 0.96 |
|  | Crude Protein (g/kg DM) | 163.33 ± 17.39 | 148.17 ± 16.20 |
|  | Crude Fiber (g/kg DM) | 241.67 ± 24.79 | 227.67 ± 24.14 |
|  | Sugar (g/kg DM) | 82.89 ± 33.64 | 71.83 ± 36.02 |
|  | Milk production (kg) | 8795.11 ± 1257.83 | 6572.20 ± 734.84 |
| Milk | Protein (%) | 3.55 ± 0.11 | 4.01 ± 1.21 |
|  | Lactose (%) | 4.51 ± 0.06 | 4.50 ± 0.08 |
|  | Fat (%) | 4.51 ± 0.21 | 4.28 ± 0.30 |
|  | Urea (mg/100 ml) | 21.44 ± 2.40 | 20.39 ± 4.52 |
|  | Bacterial Count | 31.44 ± 64.18 | 13.22 ± 15.72 |
|  | Cell Count | 129.75 ± 42.18 | 189.00 ± 80.57 |
|  | Geo Bact Count | 15.13 ± 14.94 | 17.83 ± 16.068 |
|  | Geo Cell Count | 143.88 ± 70.41 | 196.17± 59.70 |

**Table S1**: Statistical results of Kruskal-Wallis test for comparison of soil properties among the two soil types – sand and peat – and the two agriculture systems – conventional and organic. P values of ﻿﻿≤ 0.05 are considered significant.

|  | **Soil type** | | | **Agriculture system** | | |
| --- | --- | --- | --- | --- | --- | --- |
|  | Chi-squared | df | p-value | Chi-squared | df | p-value |
| N _total_ | 14.286 | 1 | **< 0.001** | 0.091 | 1 | 0.762 |
| P _avail_ | 0.092 | 1 | 0.762 | 0.013 | 1 | 0.910 |
| P _total_ | 0.824 | 1 | 0.364 | 0.116 | 1 | 0.734 |
| Organic Matter | 14.286 | 1 | **< 0.001** | 0.091 | 1 | 0.762 |
| Clay | 16.337 | 1 | **< 0.001** | 0.235 | 1 | 0.628 |
| Silt | 0.579 | 1 | 0.447 | 0.117 | 1 | 0.732 |

**Table S2**: Statistical results of Kruskal-Wallis test for comparison of use of antibiotic, ration and silage properties among the two soil types – sand and peat – and the two agriculture systems – conventional and organic. P values of ﻿﻿≤ 0.05 are considered significant.

|  |  | **Soil type** | | | **Agriculture system** | | |
| --- | --- | --- | --- | --- | --- | --- | --- |
|  |  | Chi-squared | df | p-value | Chi-squared | df | p-value |
| Ration | Antibiotics | 0.283 | 1 | 0.595 | 7.073 | 1 | **0.008** |
|  | Concentrates | 0.886 | 1 | 0.347 | 8.439 | 1 | **0.004** |
|  | Dry matter intake | 0.807 | 1 | 0.369 | 9.135 | 1 | **0.003** |
|  | Maize silage | 0.500 | 1 | 0.480 | 5.143 | 1 | **0.023** |
|  | Roughage | 4.612 | 1 | **0.032** | 4.264 | 1 | **0.039** |
|  | % grass silage | 3.090 | 1 | 0.079 | 9.135 | 1 | **0.003** |
|  | % maize silage | 0.807 | 1 | 0.369 | 9.652 | 1 | **0.002** |
|  | % concentrates | 2.812 | 1 | 0.094 | 6.851 | 1 | **0.009** |
| Silage | Dry matter | 0.240 | 1 | 0.624 | 0.500 | 1 | 0.480 |
|  | pH | 2.027 | 1 | 0.155 | 0.007 | 1 | 0.786 |
|  | Butyric acid | 0.585 | 1 | 0.445 | 2.960 | 1 | 0.085 |
|  | Acetic acid | 1.145 | 1 | 0.285 | 0.004 | 1 | 0.947 |
|  | Lactic acid | 3.500 | 1 | 0.061 | 0.161 | 1 | 0.689 |
|  | VEM | 0.135 | 1 | 0.713 | 5.556 | 1 | **0.018** |
|  | DVE | 1.222 | 1 | 0.269 | 0.503 | 1 | 0.478 |
|  | OEB | 0.304 | 1 | 0.581 | 4.762 | 1 | **0.029** |
|  | Raw Ash | 0.094 | 1 | 0.759 | 4.762 | 1 | **0.039** |
|  | OMD | 0.240 | 1 | 0.624 | 5.014 | 1 | **0.025** |
|  | NH_3_ | 0.097 | 1 | 0.756 | 2.059 | 1 | 0.151 |
|  | Nitrate | 0.867 | 1 | 0.352 | 0.347 | 1 | 0.556 |
|  | Crude Protein | 0.136 | 1 | 0.713 | 2.360 | 1 | 0.125 |
|  | Crude Fiber | 4.351 | 1 | **0.037** | 0.892 | 1 | 0.345 |
|  | Sugar | 0.376 | 1 | 0.540 | 0.170 | 1 | 0.680 |

**Table S3**: Statistical results of Kruskal-Wallis test for comparison of milk properties among the two soil types – sand and peat – and the two agriculture systems – conventional and organic. P values of ﻿﻿≤ 0.05 are considered significant.

|  | **Soil type** | | | **Agriculture system** | | |
| --- | --- | --- | --- | --- | --- | --- |
|  | Chi-squared | df | p-value | Chi-squared | df | p-value |
| Milk production | 1.131 | 1 | 0.288 | 12.370 | 1 | **< 0.001** |
| Protein | 0.240 | 1 | 0.624 | 0.963 | 1 | 0.327 |
| Lactose | 0.169 | 1 | 0.681 | 0.027 | 1 | 0.869 |
| Fat | 1.127 | 1 | 0.286 | 3.227 | 1 | 0.072 |
| Urea | 0.061 | 1 | 0.805 | 0.381 | 1 | 0.537 |
| Bacterial Count | 1.547 | 1 | 0.214 | 0.334 | 1 | 0.564 |
| Cell Count | 0.148 | 1 | 0.700 | 2.370 | 1 | 0.124 |
| Geo Bact Count | 0.496 | 1 | 0.481 | 0.707 | 1 | 0.400 |
| Geo Cell Count | 1.180 | 1 | 0.277 | 2.400 | 1 | 0.121 |

**Table S4**: Statistical results of Dunn’s test with Benjamini-Hochberg corrections for multiple comparisons of phylogenetic species variability (PSV) between sample types. P values of ﻿﻿≤ 0.05 are considered significant.

|  | | **Z-score** | **p-value** |
| --- | --- | --- | --- |
| **Fungi** | dung-milk | -1.5 | 0.099 |
|  | dung-silage | -1.2 | 0.142 |
|  | dung-soil | 3.2 | **< 0.001** |
|  | milk-silage | 0.2 | 0.410 |
|  | milk-soil | 4.7 | **< 0.001** |
|  | silage-soil | 4.2 | **< 0.001** |
| **Bacteria** | dung-milk | -2.3 | **0.011** |
|  | dung-silage | -5.1 | **< 0.001** |
|  | dung-soil | -7.7 | **< 0.001** |
|  | milk-silage | -2.8 | **< 0.001** |
|  | milk-soil | -5.5 | **< 0.001** |
|  | silage-soil | -2.7 | **< 0.001** |

**Table S5**: Most relative abundant and prevalent fungal taxa in each agriculture system in the soil, silage, milk and dung. Taxa shown per production stage are represented either by at least > 5% relative abundance in organic or conventional systems, or by > 89% prevalence in at least one of the agriculture systems.

**Table S6:** Most relative abundant and prevalent bacterial taxa in each agriculture system in the soil, silage, milk and dung. Taxa shown per production stage are represented either by at least > 5% relative abundance in organic or conventional systems, or by > 89% prevalence in at least one of the agriculture systems.

**Table S7**: Analysis of deviance for fungal community composition changes based on the relative abundances dataset within the four sample types (soil, silage, dung, milk) according to agriculture systems (conventional vs organic) and soil type (sand vs peat).

|  |  | Residual df | df | Deviance | P value |
| --- | --- | --- | --- | --- | --- |
| **Soil** | Agriculture | 17 | 1 | 1190 | 0.057 |
|  | Soil type | 16 | 1 | 1901 | **0.001** |
|  | Agric : Soil | 15 | 1 | 500 | **0.018** |
| **Silage** | Agriculture | 17 | 1 | 845.8 | 0.076 |
|  | Soil type | 16 | 1 | 1046.3 | **0.014** |
|  | Agric : Soil | 15 | 1 | 169.5 | 0.077 |
| **Dung** | Agriculture | 18 | 1 | 946.8 | **0.017** |
|  | Soil type | 17 | 1 | 974.7 | **0.006** |
|  | Agric : Soil | 16 | 1 | 211.7 | **0.023** |
| **Milk** | Agriculture | 17 | 1 | 482.1 | **0.027** |
|  | Soil type | 16 | 1 | 512.3 | **0.010** |
|  | Agric : Soil | 15 | 1 | 104.7 | **0.027** |

Multivariate test statistics: log-likelihood ratio with 999 resampling iterations via PIT-trap method. Significant results (*P* value < 0.05) are represented in bold.

**Table S8**: Analysis of deviance for bacterial community composition changes based on the relative abundances dataset within the four sample types (soil, silage, dung, milk) according to agriculture systems (conventional vs organic) and soil type (sand vs peat).

|  |  | Residual df | df | Deviance | P value |
| --- | --- | --- | --- | --- | --- |
| **Soil** | Agriculture | 17 | 1 | 1116.7 | 0.096 |
|  | Soil type | 16 | 1 | 1867 | **0.001** |
|  | Agric : Soil | 15 | 1 | 418.2 | **0.001** |
| **Silage** | Agriculture | 17 | 1 | 752.2 | 0.166 |
|  | Soil type | 16 | 1 | 741.8 | 0.118 |
|  | Agric : Soil | 15 | 1 | 307 | **0.010** |
| **Dung** | Agriculture | 18 | 1 | 940.5 | **0.011** |
|  | Soil type | 17 | 1 | 938.6 | **0.004** |
|  | Agric : Soil | 16 | 1 | 150.9 | **0.009** |
| **Milk** | Agriculture | 17 | 1 | 491.5 | **0.021** |
|  | Soil type | 16 | 1 | 496.9 | **0.004** |
|  | Agric : Soil | 15 | 1 | 91.4 | **0.001** |

Multivariate test statistics: log-likelihood ratio with 999 resampling iterations via PIT-trap method. Significant results (*P* value < 0.05) are represented in bold.

**Table S9:** Analysis of deviance for fungal and bacterial community composition differences in dung and milk, based on both the presence-absence ‘(01)’and the relative abundances ‘(ab)’ datasets, in relation to the content of ration given to the cows.

| **Dung fungi (01)** |  |  |  |  |  | **Dung bacteria (01)** | |  |  |  |
| --- | --- | --- | --- | --- | --- | --- | --- | --- | --- | --- |
|  | Res. Df | Df. Diff | Dev | P |  |  | Res. Df | Df. Diff | Dev | P |
| (Intercept) | 18 |  |  |  |  | (Intercept) | 16 |  |  |  |
| % grass | 17 | 1 | 1049 | 0.920 |  | % grass | 15 | 1 | 761.2 | 0.192 |
| Concentrates | 16 | 1 | 1018 | 0.390 |  | Concentrates | 14 | 1 | 839.4 | 0.157 |
| Antibiotics | 15 | 1 | 1436 | 0.002** |  | Antibiotics | 13 | 1 | 830.3 | 0.382 |
|  |  |  |  |  |  |  |  |  |  |  |
| **Dung fungi (ab)** |  |  |  |  |  | **Dung bacteria (ab)** | |  |  |  |
|  | Res. Df | Df. Diff | Dev | P |  |  | Res. Df | Df. Diff | Dev | P |
| (Intercept) | 17 |  |  |  |  | (Intercept) | 16 |  |  |  |
| % grass | 16 | 1 | 641.7 | 0.041* |  | % grass | 15 | 1 | 733.3 | 0.135 |
| Antibiotics | 15 | 1 | 517.4 | 0.236 |  | Concentrates | 14 | 1 | 697.9 | 0.218 |
| Concentrates | 14 | 1 | 336.6 | 0.094 |  | Antibiotics | 13 | 1 | 751.1 | 0.394 |
|  |  |  |  |  |  |  |  |  |  |  |
| **Milk fungi (01)** |  |  |  |  |  | **Milk bacteria (01)** | |  |  |  |
|  | Res. Df | Df. Diff | Dev | P |  |  | Res. Df | Df. Diff | Dev | P |
| (Intercept) | 17 |  |  |  |  | (Intercept) | 16 |  |  |  |
| Antibiotics | 16 | 1 | 641.7 | 0.010* |  | Antibiotics | 15 | 1 | 1380 | 0.267 |
| Concentrates | 15 | 1 | 517.4 | 0.026* |  | % grass | 14 | 1 | 1418 | 0.409 |
| % grass | 14 | 1 | 336.6 | 0.035* |  | Concentrates | 13 | 1 | 1741 | 0.091 |
|  |  |  |  |  |  |  |  |  |  |  |
| **Milk fungi (ab)** |  |  |  |  |  | **Milk bacteria (ab)** | |  |  |  |
|  | Res. Df | Df. Diff | Dev | P |  |  | Res. Df | Df. Diff | Dev | P |
| (Intercept) | 17 |  |  |  |  | (Intercept) | 16 |  |  |  |
| Antibiotics | 16 | 1 | 798.2 | 0.037* |  | Antibiotics | 15 | 1 | 1388 | 0.230 |
| Concentrates | 15 | 1 | 984.6 | 0.045* |  | % grass | 14 | 1 | 1220 | 0.320 |
| % grass | 14 | 1 | 1058.4 | 0.352 |  | Concentrates | 13 | 1 | 1454 | 0.093 |

**Table S10:** Analysis of deviance for fungal and bacterial community composition changes in the milk, based on both the presence-absence ‘(01)’ and the relative abundances ‘(ab)’ datasets, in relation to milk properties.

| **Milk fungi (01)** |  |  |  |  |  | **Milk bacteria (01)** | |  | |  | |  | |  | |
| --- | --- | --- | --- | --- | --- | --- | --- | --- | --- | --- | --- | --- | --- | --- | --- |
|  | Res. Df | Df. Diff | Dev | P |  |  | Res. Df | | Df. Diff | | Dev | | P | |
| (Intercept) | 17 |  |  |  |  | (Intercept) | 16 | |  | |  | |  | |
| Milk productivity | 16 | 1 | 595.3 | 0.023* |  | Milk productivity | 15 | | 1 | | 1413 | | 0.195 | |
| Fat | 15 | 1 | 504.4 | 0.016* |  | Urea | 14 | | 1 | | 1777 | | 0.038* | |
| Urea | 14 | 1 | 320.5 | 0.029* |  | Fat | 13 | | 1 | | 1926 | | 0.015* | |
|  |  |  |  |  |  |  |  | |  | |  | |  | |
| **Milk fungi (ab)** |  |  |  |  |  | **Milk bacteria (ab)** |  | |  | |  | |  | |
|  | Res. Df | Df. Diff | Dev | P |  |  | Res. Df | | Df. Diff | | Dev | | P | |
| (Intercept) | 17 |  |  |  |  | (Intercept) | 16 | |  | |  | |  | |
| Milk productivity | 16 | 1 | 1040 | 0.006** |  | Milk productivity | 15 | | 1 | | 1513 | | 0.112 | |
| Fat | 15 | 1 | 900.7 | 0.061 |  | Urea | 14 | | 1 | | 1536 | | 0.230 | |
| Urea | 14 | 1 | 632.9 | 0.509 |  | Fat | 13 | | 1 | | 2015 | | 0.068 | |

# Figures

**Figure S1**: Rarefaction curves obtained for total number of Zotus obtained in the four production stages for the fungi (a) and bacteria (b) datasets. Solid and dashed lines represent the rarefied and extrapolated number of Zotus per sample, calculated using the R package iNEXT. Dots represent the true number of reads obtained; when dots are absent, the total number of reads is larger than shown in the plots. Vertical lines represent the threshold used for resampling (2500 for fungi and 1000 for bacteria).

**Figure S2**: Histograms of the linear discriminant analysis (LDA) scores computed for Bacteria (top panel) and Fungi (low panel) differentially abundant between organic and conventional agriculture systems. LEfSe calculates LDA scores which can be interpreted as the degree of consistent difference in relative abundance of specific taxa at different taxonomic levels between the two farming types (classes), considering the two soil types (sub-classes).

**Figure S3**: Fungal and bacterial communities’ structure in the soil (a), silage (b), dung (c), and milk (d), colored according to agriculture systems. Fungi are represented as empty symbols and bacteria with full symbols.
